# Infantile Inflammatory Bowel Disease in a Three-Month-Old-Boy

**DOI:** 10.7759/cureus.12743

**Published:** 2021-01-16

**Authors:** Chie Iida, Ako Tatsumi, Hisanori Fujino, Kaori Anzai, Shinichi Sumimoto

**Affiliations:** 1 Pediatrics, Osaka Red Cross Hospital, Osaka, JPN

**Keywords:** colonoscopy, inflammatory bowel disease, monogenic ibd, milk allergy, steroid, mesalazine

## Abstract

Very early-onset inflammatory bowel disease (VEO-IBD) and infantile IBD occur in children aged less than six years and less than two years, respectively. Since childhood-onset IBD seems to be a more aggressive and rapidly progressive disease than adult-onset IBD, it should therefore be diagnosed and treated immediately. Here, we report a case of infantile IBD in a three-month-old infant with clinical and biochemical manifestations. The diagnosis was confirmed with histopathological evidence. The patient had been treated successfully with both mesalazine and prednisolone and with mesalazine alone on follow-up.

## Introduction

Very early-onset inflammatory bowel disease (VEO-IBD) occurs before the age of six years and infantile IBD occurs before the age of two years [1-2]. The clinical characteristics of VEO-IBD or infantile IBD are different from those typically observed in adolescent- or adult-onset IBD [3]. The features of childhood-onset IBD are typically more aggressive and rapidly progressive as compared to the characteristics of the adult cohort [2,4-5]. The IBD diagnosed in childhood may also have a strong genetic component [6-7]. VEO-IBD associated with single genetic mutations is classified as monogenic IBD [8], and approximately 50 genetically different mutations have been identified using advanced genetic sequencing techniques [9-10]. IBD should be considered in the differential diagnosis of any child with persistent diarrhea, hematochezia, failure to thrive, and/or poor feeding. We report the case of a three-month-old Japanese boy who presented with diarrhea and hematochezia and was finally diagnosed as VEO-IBD or infantile IBD and treated. He showed good improvement.

## Case presentation

The patient presented to our hospital for evaluation and treatment of hematochezia and poor feeding. He had diarrhea for approximately 1 month and hematochezia for 14 days duration; he was treated with probiotics for 7 days without improvement. He had no past medical history and relevant family history. He had age-appropriate vaccinations, such as Prevenar 13 suspension liquid for injection (Pfizer, Brooklyn, New York), ActHIB (Sanofi S.A., Paris, France), Bimmugen (KM Biologics, Kumamoto, Japan), and Rotarix (GlaxoSmithKline plc, Brentford, England). Also, the development was appropriate as per his age. He was formula-fed since birth. The boy was physically examined, and the results revealed that he was healthy, active, pale, had no dysmorphic features, and had adequate hydration. His abdominal examination had been normal with no anal fissures. The rest of the examination was unremarkable.

**Figure 1:**
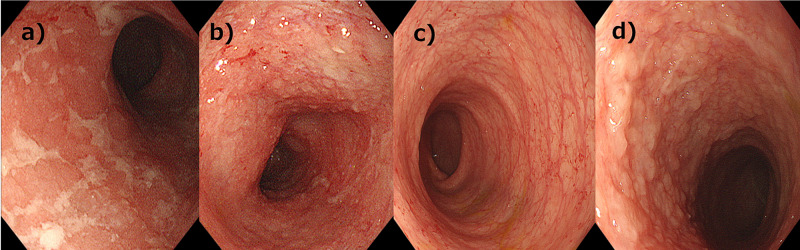
Results from colonoscopy 1 a,b) Colonoscopy revealed ulcers and pus in the rectum and descending colon, and loss of mucous membranes from the rectum to the middle of the transverse colon. 1 c,d) Results from colonoscopy improved after one month of treatment.

The laboratory results revealed the following: white blood cell count (WBC) 20970/μL, with no eosinophils; hemoglobin 10.2 g/dL; C-reactive protein (CRP) 0.76 mg/dL; albumin 3.3 g/dL; and total immunoglobulin G (IgG) 116 mg/dL. We administered intravenous fluids while he was under observation for suspected milk allergy or lymphoid follicular proctitis. By the third day of hospitalization, his condition had deteriorated: WBC 12,270 /μL; hemoglobin 7.9 g/dL; CRP 0.69 mg/dL; and albumin 2.2 g/dL. An abdominal ultrasound revealed edema in the descending and transverse colon. The patient was fed with casein hydrolysate milk. On the seventh day of hospitalization, his symptoms persisted and his laboratory values continued to deteriorate: WBC 24,510/μL; hemoglobin 9.1 g/dL; CRP 0.54 mg/dL; albumin 2.4 g/dL; and total IgG 102 mg/dL; conversely, his immunological screening results were normal. We suspected severe milk allergy and changed his formula from casein hydrolysate milk to amino-acid-based milk. However, no improvements were seen in his symptoms with an amino acid-based formula, so we initiated fasting. On the fourteenth day of hospitalization, a colonoscopy revealed ulcers and pus in the rectum and descending colon associated with loss of mucous membrane from the rectum to the middle of the transverse colon (Figure 1, panels 1a-1b). We ruled out cytomegalovirus (CMV) infection, food protein-induced enterocolitis syndrome (FPIES), and primary immunodeficiency disorders based on the laboratory findings (Table 1). The histopathology report of the intestine revealed crypt architectural abnormalities and apoptosis, strongly suggesting IBD. However, plasmacytosis of the lamina propria of the mucous membrane was not so severe based on the macroscopic findings, which strongly suggested monogenic IBD. Thus, the patient was diagnosed with VEO-IBD.

**Table 1:** Blood test result Ruled out cytomegalovirus (CMV) infection, food protein-induced enterocolitis syndrome (FPIES), primary immunodeficiency disorders

【allergy】		【immunology】	
ALST		CD8	16.5%
κcasein	negative(SI=0.55)	CD4	56.8%
lactoferrin	positive(SI=4.18)	CD3	71.1%
lactalbumin	negative(SI=1.03)	CD20	22.2%
【infection】		CD4/8ratio	3.4
CMV IgM(EIA)	negative	PHA lymphocyte transformation	95CPM
CMV IgG(EIA)	negative	Con-A lymphocyte transformation	95CPM
T-SPOT	negative	neutrophil bactericidal activity	97.25%
【IgG subclass】		neutrophil phagocytosis activity	96.93%
IgG1	66.84%	antinuclear antibody	<1:40
IgG2	12.33%	MPO-ANCA	<1.0U/mL
IgG3	18.76%	PR3-ANCA	<1.0U/mL
IgG4	<2.07%		

**Figure 2:**
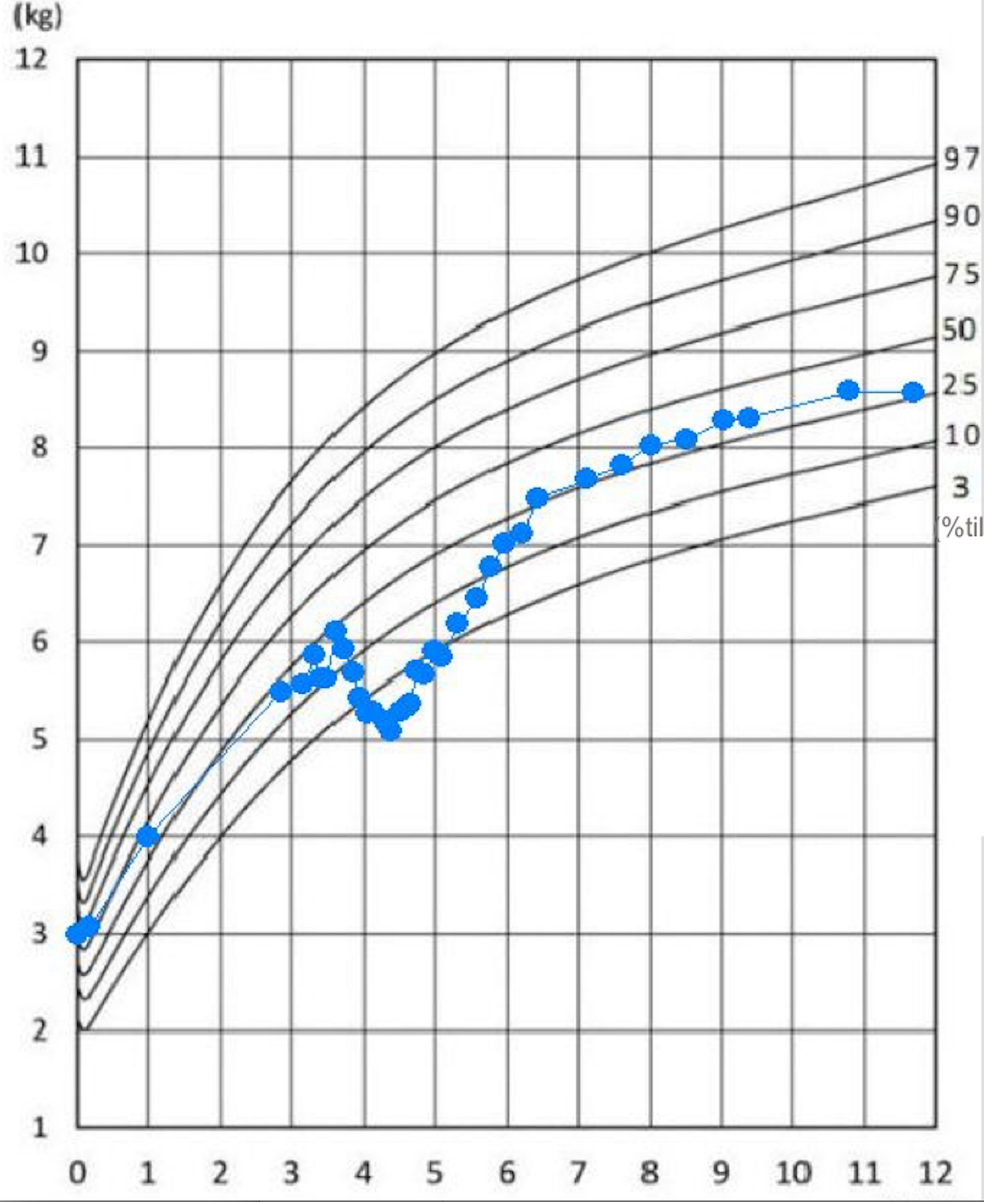
Infant body weight curve The infant's body weight increased after appropriate treatment.

**Figure 3:**
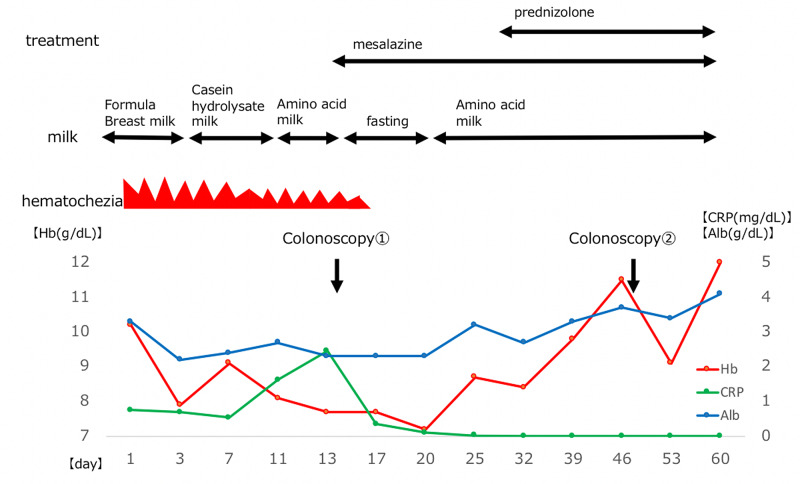
Clinical course

The patient’s symptoms and laboratory findings improved in response to the treatment with mesalazine and prednisolone for one month; no relapse was observed after steroid taper. Results from a follow-up colonoscopy performed one month later also revealed remarkable improvement, hence the steroid treatment was discontinued (Figure 1, panels 1c-1d). At 18 months old, his clinical condition was under control with mesalazine only. His physical development was appropriate for his age (Figure 2). Whole-exome sequencing did not detect any significant mutations in IBD-associated genes (Figure 3).

## Discussion

The incidence of pediatric IBD is increasing worldwide [11-12], although neonatal or infantile-onset IBD develops in less than 1% of the pediatric population [13]. Treatment approaches for pediatric patients sometimes differ from those for adult patients. To date, all effective therapies in adults have also been effective in children. This is a great need for clinical trials of new therapies in children so that they have equal access to emerging treatments and optimal pediatric dosing can be established [14].

Our patient experienced severe diarrhea and hematochezia; the differential diagnosis included infection, milk allergy, and lymphoid follicular hyperplasia, although all tests for these disorders were negative. Colonoscopy and pathological findings of intestine specimens confirmed the diagnosis of VEO-IBD, and treatment with mesalazine and steroids was effective at promoting resolution. We initially administered mesalazine 50 mg/kg/day and prednisolone 1 mg/kg/day. Long-term steroid use is not preferable for pediatric patients due to its side effects such as disturbance in growth, osteoporosis, and diabetes. Hence, steroid use is recommended only for a short period in children. When these treatments are deemed ineffective, immunosuppressive therapy or stem cell transplantation should be considered as an alternative.

In this case, no significant mutations in any of the genes known to be associated with VEO-IBD were detected. Approximately 50 genetically different mutations have been identified with monogenic IBD using advanced genetic sequencing techniques. With further research, new biomarkers and advanced diagnostic techniques used in the field of gastrointestinal endoscopy, molecular pathology, and genetics need to be developed for establishing appropriate diagnosis and treatment.

## Conclusions

In conclusion, VEO-IBD should be included in the differential diagnosis of pediatric patients presenting with persistent diarrhea and hematochezia, and colonoscopy should be performed as soon as possible when VEO-IBD is suspected in order to facilitate timely treatment.
